# A cross-sectional study of knowledge and practices in the management of patients with Parkinson’s disease amongst public practice-based general practitioners and geriatricians

**DOI:** 10.1186/s12913-022-07503-7

**Published:** 2022-01-20

**Authors:** Isis Claire Z. Y. Lim, Seyed Ehsan Saffari, Shermyn Neo

**Affiliations:** 1 Department of Medicine, Yong Loo Lin School of Medicine, National University of Singapore, Singapore, Singapore; 2 Centre for Quantitative Medicine, Duke-NUS Medical School, National University of Singapore, Singapore, Singapore; 3 Department of Neurology, National Neuroscience Institute, Singapore, Singapore

**Keywords:** Parkinson’s disease, General practitioner, Geriatrician, Quality measures

## Abstract

**Background:**

As most patients are likely to first interface with their community general practitioner (GP) or geriatrician for chronic healthcare conditions, these non-neurologists practitioners are well-placed to diagnose, initiate treatment in symptomatic Parkinson’s disease (PD) patients, and provide regular and timely management of their PD. However, current studies suggest that the role of the GP and geriatrician in providing holistic care for PD patients may be limited by factors such as patient perceptions, and a lack of knowledge base in the quality measures of care. This paper aims to better understand the different management styles between GPs and geriatricians practicing in public institutions in Singapore, qualify the difficulties they face in providing patient-centric care for PD patients, and identify any gaps in quality measures of care.

**Methods:**

A questionnaire was completed anonymously by GPs (*n* = 43) and geriatricians (*n* = 33) based at public institutions, on a voluntary basis before a compulsory didactic teaching on PD. Questions were modelled after quality measures set out by the American Academy of Neurology, specifically eliciting information on falls, non-motor symptoms, exercise regime and medication-related symptoms. “PD management practices and styles” questions were answered by the respondents on a 4-point Likert scale.

**Results:**

Geriatricians spent more time in consult with PD patients compared with GPs (median [Q1-Q3] = 20 [15–30] vs 10 [10–15] minutes, *p* <  0.001). Geriatricians were more comfortable initiating PD medications than GPs (OR = 11.8 [95% CI: 3.54–39.3], *p* <  0.001), independent of gender, years of practice and duration of consult. Comfort in initiating dopamine replacement therapy (OR 1.06 [1.00–1.36], *p* = 0.07; aOR = 1.14 [1.02–1.26], *p* = 0.02) also increased with physician’s years of practice. Unfamiliarity with the types and/or doses of the medications was the most cited barrier faced by GPs (76.7%). Geriatricians were more likely than GPs to ask about falls (100% vs 86.0%, *p* = 0.025), non-motor symptoms (75.8% vs 53.5%, *p* = 0.049) and the patient’s regular physical activities (72.7% vs 41.9%, *p* = 0.01).

**Conclusions:**

This study identified key patterns in the management practices and styles of non-neurologists physicians, and identified gaps in current practice. Our data suggests that interventions directed at education on PD medication prescriptions and provision of patient PD education, creation of best clinical practice guidelines, and accreditation by national bodies may instil greater confidence in practitioners to initiate and continue patient-centric PD care. A longer consultation duration with PD patients should be considered to allow physicians to get a greater scope of the patient’s needs and better manage them.

**Supplementary Information:**

The online version contains supplementary material available at 10.1186/s12913-022-07503-7.

## Introduction

Considering the diverse presentations of motor and non-motor signs and symptoms in Parkinson’s disease (PD) patients, a systematic approach is required to diagnose and ensure holistic care of the patient [1, 2].

In many countries, the majority of patients are likely to first interface with their community general practitioner (GP) or their geriatrician for chronic healthcare conditions [3], which often leaves the GP or geriatrician to be the first to recognise the symptoms of early PD or initiate PD treatment. However, few studies have been done to investigate how GPs and geriatricians manage PD in their clinics and whether they refer to or communicate with a neurologist regarding their treatment plans for these patients.

The prevalence of PD in Singapore is 0.3% in those aged 50 and above; with a resident population of 3.99 million in 2021, we estimate that there are close to 12,000 PD patient [4]. In Singapore, GPs and geriatricians are often the first point of contact for patients. There are specialized Geriatric Medicine (GRM) departments in all 7 major hospitals in Singapore, with a total of 132 registered geriatricians as of 2020 with the majority practicing in public institutions, compared to 111 registered neurologists. Geriatricians undergo a 3-year residency to qualify as a specialist and are trained to manage complex medical conditions in the elderly population, in particular the 5 giants of geriatrics – iatrogenesis, immobility, instability, incontinence and impaired cognition [5], which are problems commonly encountered in PD patients, who are also more likely to be elderly. They may act as the patient’s primary physician or may refer them to a specialist for a second opinion, if the case is expected to be more complex. There are 22 polyclinics (public healthcare centers that provide subsidized primary care) in the country, spread out evenly among each neighborhood. Compared to geriatricians, structured training is not compulsory for GPs, and the physicians at the polyclinics may or may not have undergone a Family Medicine residency program (3-year long), or a graduate diploma in Family Medicine (24-month part-time course). In the 2014 Primary Care Survey conducted by the Singapore Ministry of Health, they reported that of the 18 polyclinics available at the time, and 1409 private GP clinics in Singapore, polyclinics had a market share of 20% of the total medical attendees per day [6]. In the community, polyclinic GPs manage about 45% of patients with chronic medical illnesses [7], and are therefore well-positioned to recognize the symptoms of PD and manage it within their capacity or refer to a specialist for further management.

As patients often wait for long periods in between their neurology consults, thus the ideal situation would be for the GPs and geriatricians to initiate treatment in symptomatic PD upon diagnosis to prevent disability, or to contact the neurologist caring for the patient should they discover a complication throughout the disease course [8]. Studies have shown that PD patients who received neurologist care had improved survival demonstrated by higher survival rates and an increase in odds of survival compared with primary care physician–treated subjects [9]. Therefore, it is important to identify the difficulties faced by non-neurologist physicians in the care of PD patients in the community to narrow the gaps in their care.

This paper aims to better understand the level of confidence diagnosing and treating PD and the management styles of GPs and geriatricians practicing in public institutions in Singapore, qualify the difficulties they face in providing patient-centric care for PD patients, as well as identify any gaps in quality measures of care.

## Methods

### Study design

We conducted a cross-sectional survey of knowledge and management practices in PD amongst physicians from 6 polyclinics that served the central and northern regions of Singapore, and 2 GRM departments in large tertiary hospitals in Singapore, before a didactic teaching session on PD. These teaching sessions were conducted over a period of 2 years from 2019 to 2020.

### Recruitment

Didactic sessions on PD were held for GPs and geriatricians, as part of compulsory continuing medical education (CME), and all attendees were requested to complete a questionnaire on their experiences with managing PD patients before the sessions. The GP teaching was conducted online via Zoom, and delivered to GPs from 6 polyclinics. It was opened to all physicians in the 6 polyclinics and an audience size of 150 was anticipated. The GRM teaching was held in person, and delivered to 2 GRM departments in tertiary hospitals in Singapore. Both trainees in the GRM residency and consultant physicians in the department were invited for these sessions; department strength ranged from 20 to 30 consultants and 5–13 residents.

### Questionnaire

The items in the questionnaire were designed to gather information on the confidence of GPs and GRMs in diagnosing and initiating treatment in PD, their comfort level in prescribing PD medications, barriers to PD treatment, as well as their PD management practices and styles, to guide the objectives of the talk (Additional file 1). For “reasons for discomfort starting dopamine replacement therapy”, participants could choose all the options that applied to them. Questions on PD management practices were modeled after quality measures set out by the American Academy of Neurology (AAN) in 2015 – namely measures 3–6 in the AAN recommendations were consolidated into a question on non-motor symptoms in PD, while measure 7 on falls, measure 9 on regular exercise regime, and measure 10 on medication-related motor symptoms were posed as individual questions [10]. ‘PD management practices and styles’ questions were answered by the respondents on a 4-point Likert scale ranging from 1 (I almost always do this) to 4 (I rarely do this).

A quantitative study design was chosen to cover a greater breadth of differences between the challenges and practices of GPs and geriatricians. Respondents were given an optional open-ended question to provide in-depth insight on the barriers to initiating PD treatment.

The questions were designed by SN, a practicing movement disorder specialist at the National Neuroscience Institute of Singapore. An initial 7-item questionnaire was piloted amongst GPs attending an in-person talk on PD at a polyclinic (not included in the final cohort) and subsequently modified for clarity based on the responses and feedback.

Prior to the commencement of the talks, the finalized questionnaire was sent out via a Google form link to the participants, and was filled up anonymously on a voluntary basis.

### Statistical analysis plan

Data analysis was performed using IBM SPSS version 27 (IBM Corp. Released 2020. IBM SPSS Statistics for Windows, Version 27.0. Armonk, NY: IBM Corp). Descriptive analysis was conducted for sociodemographic variables. Median and first- and third-quartiles (Q1 and Q3) were reported for continuous variables while frequencies and percentages were presented for categorical variables. Responses to Likert scale questions were re-coded to differentiate between two levels of agreement: (1) “almost always” or “often”, and (2) “sometimes” or “rarely”. Comparisons between GPs and geriatricians were made using Mann-Whitney U test and Pearson’s chi-square for continuous and categorical variables, respectively. Logistic regression analysis was performed to investigate the factors affecting PD diagnosis and treatment, adjusting for potential confounders including gender, number of years of practice, and duration spent with patients per consultation. Firth’s penalized likelihood approach was applied to adjust the odds ratio and 95% confidence intervals and reduce the bias in the presence of imbalanced variable proportions. Similar methods were conducted to study the factors affecting PD care quality measures and PD management practices and styles.

Variables included in the multivariable regression model were those that significantly differed amongst GPs and geriatricians, as well as those selected a priori based on previously identified factors that influenced physician practices. Female doctors have been shown to provide more preventive services and psychosocial counselling compared to their male counterparts, whereas male doctors may spend more time on technical practice behaviors such as medical history taking [11]. Consultation length was included as systematic reviews have shown that increased consultation length improves doctor-patient communication and quality of care [12, 13]. Lastly, the duration of physician’s clinical practice was included as a systemic review done showed consistent positive associations between patient experience, patient safety and clinical effectiveness for a wide range of diseases [14]. Significance level was set at *p* value < 0.05.

As the questionnaire was designed with the aim of quality measurement and improvement, and did not collect any identifiable data, the study was exempt from ethics review as per our institutional review board guidelines.

## Results

### General respondent characteristics

A total of 43 GPs and 33 geriatricians responded to the questionnaire, of whom, 51.2% of GPs (*n* = 22) and 33.3% of geriatricians (*n* = 11) were male (*p* = 0.12). The survey response rate was 28.7% amongst GPs and 48.5% amongst geriatricians (*p* <  0.001). Median [Q1-Q3] duration of practice was similar in both groups – 9 years [6–16.5] in the GP group and 7 years [5–13] in the GRM group.

Geriatricians spent longer time in consults with PD patients than GPs (median [Q1-Q3] = 20 min [15–30] vs 10 min [10–15], *p* <  0.001). Table 1 summarizes the questionnaire responses according to the medical specialty of the respondents.

**Table 1:** Questionnaire responses according to medical specialty

Item	GP (*n* = 43)	Geriatrician (*n* = 33)	*p* value
Demographic data			
Male, n (%)	22 (51.2%)	11 (33.3%)	0.12
Years of practice, median (Q1-Q3)	9 (6–16.5)	7 (5–13)	0.74
Consult duration in minutes, median (Q1-Q3)	10 (10–15)	20 (15–30)	**< 0.001**
PD diagnosis and treatment, n(%)			
Confident making PD diagnosis	30 (69.8%)	26 (78.8%)	0.38
Comfortable starting DRT	4 (9.3%)	19 (57.6%)	**< 0.001**
Reasons for discomfort starting DRT			
Not familiar with types and/or dosage of medications)	33 (76.7%)	10 (30.3%)	< 0.001
Not comfortable with providing PD education)	27 (62.8%)	5 (15.2%)	< 0.001
Patients want a neurologist’s diagnosis of PD before starting medication)	19 (44.1%)	3 (9.10%)	0.002
PD management practices and styles, n(%)			
Query about falls	37 (86.0%)	33 (100%)	**0.025**
Query about non-motor symptoms	23 (53.5%)	25 (75.8%)	**0.049**
Query about side effects of PD medications	24 (55.8%)	24 (72.7%)	0.13
Query about medication timing in relation to meals	10 (23.2%)	11 (33.3%)	0.33
Query about physical activity	18 (41.9%)	24 (72.7%)	**0.01**
Refer to neurologist	42 (97.7%)	4 (12.1%)	**< 0.001**
Contact neurologist before initiating change in medications	8 (18.6%)	10 (30.3%)	0.23
Correctly identified whether department had guidelines for PD care	13 (30.2%)	17 (51.5%)	0.06

*GP* general practitioner, *IQR* interquartile range, *PD* Parkinson’s Disease, *DRT* dopamine replacement therapy; Q1 and Q3, first- and third-quartile

### PD diagnosis and treatment practices

The majority of respondents in both geriatricians and GPs groups reported having confidence in making the diagnosis of PD, with no significance difference noted (69.8% of GPs and 78.8% of geriatricians, *p* = 0.38) (Table 2).

**Table 2:** Univariate and multivariable logistic regression analysis of factors affecting PD diagnosis and treatment

	Confidence in diagnosis				Confidence in starting DRT				Unfamiliarity with medication				Discomfort in provision of PD education				Patients want a specialist opinion			
	OR (95% CI)	*p* value	aOR (95% CI)	*p* value	OR (95% CI)	*p* value	aOR (95% CI)	*p* value	OR (95% CI)	*p* value	aOR (95% CI)	*p* value	OR (95% CI)	*p* value	aOR (95% CI)	*p* value	OR (95% CI)	*p* value	aOR (95% CI)	*p* value
**Medical specialty**																				
GP	Ref.								7.14 (2.58,19.8)	**< 0.001**	5.75 (1.33,24.91)	**0.019**	8.64 (2.84,26.3)	**< 0.001**	8.43 (1.62,43.9)	**0.011**	6.94 (1.94,24.8)	**0.003**	7.64 (1.13,51.5)	**0.037**
Geriatrician	1.61 (0.56,4.64)	0.38	1.20 (0.23,6.22)	0.83	11.8 (3.54,39.3)	**< 0.001**	8.80 (1.65,47)	**0.011**	Ref.											
**Gender**																				
Male	Ref.																			
Female	2.50 (0.88,7.11)	0.09	2.60 (0.82,8.29)	0.11	0.77 (0.29,2.07)	0.61	0.69 (0.16,2.88)	0.61	1.16 (0.46,2.89)	0.75	1.54 (0.47,5.03)	0.47	0.50 (0.20,1.27)	0.15	0.55 (0.18,1.73)	0.31	0.53 (0.20,1.44)	0.22	0.63 (0.20,2.02)	0.44
**Years of practice**	1.07 (0.99,1.16)	0.10	1.08 (0.99,1.18)	0.08	1.06 (1.00,1.12)	0.07	1.14 (1.02,1.26)	**0.02**	0.96 (0.91,1.01)	0.13	0.92 (0.85,1.00)	**0.049**	0.95 (0.89,1.01)	0.09	0.91 (0.83,0.99)	**0.02**	0.94 (0.87,1.01)	0.09	0.90 (0.82,0.99)	**0.03**
**Duration spent with patient (min)**	1.03 (0.95,1.12)	0.44	1.03 (0.92,1.15)	0.63	1.13 (1.05,1.23)	**0.002**	1.09 (0.97,1.22)	0.17	0.90 (0.83,0.97)	**0.007**	0.96 (0.86,1.06)	0.40	0.91 (0.83,0.98)	**0.02**	1.00 (0.90,1.12)	0.95	0.92 (0.84,1.00)	0.06	1.01 (0.89,1.15)	0.85

*PD* Parkinson’s Disease, *GP* general practitioner, *OR* odds ratio, *aOR* adjusted odds ratio

Geriatricians were more comfortable in initiating dopamine replacement therapy (DRT) for patients they had diagnosed with PD, as compared to GPs (OR = 11.8 [95% CI: 3.54–39.3], *p* <  0.001). When asked further about why they may not feel comfortable with starting the treatment, GPs were more likely to report that they were unfamiliar with the types and/or doses of PD medications as compared to geriatricians (OR 7.14 [2.58–19.8], *p* <  0.001). GPs were more likely to report being uncomfortable with providing PD education (OR 8.64 [2.84–26.3], p <  0.001), and their patients were more likely to request a neurologist confirmation before initiation of the treatment (OR 6.94 [1.94–24.8], *p* = 0.003).

Participants were given an option in this question to include free text responses. Notably, 2 participants shared that they were unsure about the treatment indications and treatment thresholds, and 1 shared that it was important to have a multidisciplinary team consisting of physiotherapy and rehabilitation to provide better care to the patient.

After adjusting for gender, number of years of practice, and duration of time spent with patient per consult in our multivariable regression model, we found that being a geriatrician remained significantly associated with higher confidence in starting DRT (adjusted OR (aOR) 8.80 [1.65–47.0], *p* = 0.011). Conversely, being a GP was associated with unfamiliarity with PD medications (aOR 5.75 [1.33–24.9], *p* = 0.019), discomfort in provision of PD education (aOR 8.43 [1.62–43.9], p = 0.011), and a higher likelihood of encountering patients who would want a neurologist opinion before initiating PD treatment (aOR 7.64 [1.13–51.5], *p* = 0.037).

In addition, greater number of years of practice was inversely related to the physician’s unfamiliarity with types and doses of PD medications (aOR 0.92 [0.85–1.00], *p* = 0.049) and discomfort in providing PD education (aOR 0.91 [0.83–0.99], *p* = 0.02). Physicians with more years of experience were also less likely to report that their ‘patients would want a neurologist confirmation of the diagnosis before initiating PD treatment’ as a barrier to initiating DRT, aOR 0.90 [0.82–0.99], *p* = 0.03 (Table 2).

To better understand the prescription patterns of GPs and geriatricians, respondents were also asked to rank the PD medications in the order of comfort - with 1 being the most comfortable, and 5 being the least comfortable. Both GPs and geriatricians stated that the average order of comfort with regards to medications were: levodopa (most comfortable), dopamine agonist, selegiline, amantadine, and trihexyphenidyl (least comfortable).

### PD management practices and styles

Table 3 summarizes the univariate and multivariable analysis of factors affecting PD care quality measures. During consults, geriatricians were more likely than their GP counterparts to ask about falls (100% vs 86%, *p* = 0.025), non-motor symptoms (OR 2.72 [1.00–7.36], *p* = 0.049), physical activity (OR 3.70 [1.40–9.84], *p* = 0.01). After correction for gender, number of years of practice and duration spent with patient per consult, queries on PD medications side effects was the only significant practice associated with being a geriatrician (aOR 4.63 [1.04–20.6], p = 0.04). Both GPs and geriatricians did not ask regularly about medication timing in relation to meals (23.2% of GPs and 33.3% of geriatricians, *p* = 0.33).

**Table 3:** Univariate and multivariable logistic regression analysis on PD care quality measures

	Falls				Non-motor symptoms				Side effects of PD medications				Medication timing in relation to meals				Physical activity			
	OR (95% CI)	*p* value	aOR (95% CI)	*p* value	OR (95% CI)	*p* value	aOR (95% CI)	*p* value	OR (95% CI)	*p* value	aOR (95% CI)	*p* value	OR (95% CI)	*p* value	aOR (95% CI)	*p* value	OR (95% CI)	*p* value	aOR (95% CI)	*p* value
**Medical specialty**																				
GP	Ref																			
Geriatrician	–	–	–	–	2.72 (1.00,7.36)	**0.049**	2.31 (0.59,8.96)	0.23	2.11 (0.80,5.59)	0.13	4.63 (1.04,20.6)	0.04	1.65 (0.60,4.54)	0.33	1.50 (0.37,6.10)	0.57	3.70 (1.40,9.84)	**0.01**	3.26 (0.83,12.8)	0.09
**Gender**																				
Male	Ref																			
Female	1.33 (0.25,7.08)	0.74	1.08 (0.18,6.36)	0.93	0.96 (0.38,2.47)	0.94	0.81 (0.30,2.22)	0.68	1.21 (0.48,3.10)	0.69	1.16 (0.42,3.18)	0.77	0.79 (0.29,2.17)	0.65	0.69 (0.23,2.05)	0.51	1.31 (0.53,3.26)	0.57	0.98 (0.36,2.72)	0.98
**Years of practice**	0.97 (0.89,1.06)	0.51	0.97 (0.86,1.09)	0.57	0.99 (0.94,1.04)	0.70	0.99 (0.94,1.05)	0.81	1.02 (0.96,1.08)	0.52	1.02 (0.95,1.08)	0.62	1.01 (0.96,1.07)	0.68	1.01 (0.95,1.08)	0.76	1.02 (0.97,1.08)	0.50	1.03 (0.96,1.09)	0.45
**Duration spent with patient (min)**	1.09 (0.92,1.27)	0.32	0.89 (0.68,1.15)	0.37	1.06 (0.98,1.15)	0.13	1.02 (0.92,1.13)	0.73	0.99 (0.92,1.06)	0.73	0.92 (0.83,1.02)	0.11	1.04 (0.97,1.11)	0.31	1.02 (0.93,1.13)	0.63	1.09 (1.01,1.18)	**0.04**	1.03 (0.93,1.14)	0.57

*PD* Parkinson’s Disease, *GP* general practitioner, *OR* odds ratio, *aOR* adjusted odds ratio

Table 4 summarizes the univariate and multivariable analysis of factors affecting PD management practices and styles. In the univariate analysis, GPs were more likely to refer a patient they had diagnosed with PD to a neurologist for further management, (OR 185.7 [28.0 – > 999], *p* <  0.001). After correction for gender, number of years of practice, duration spent with patient per consult, GPs were shown to have significant association with referrals to neurologists (aOR 306 [12.6 – > 999], p <  0.001). Both GPs and geriatricians reported low rates of contacting the patient’s managing neurologist (GPs 18.6% and geriatricians 30.3%, *p* = 0.23).

**Table 4:** Univariate and multivariable logistic regression analysis on PD management practises and styles

	Refer to neurologist				Contact neurologist before initiating change in medications			
	OR (95% CI)	*p* value	aOR (95% CI)	*p* value	OR (95% CI)	*p* value	aOR (95% CI)	*p* value
**Medical specialty**								
GP	185.7 (28, > 999)	< 0.001	306 (12.6, > 999)	< 0.001	Ref.			
Geriatrician	Ref.				1.90 (0.65,5.54)	0.24	4.50 (0.82,24.6)	0.08
**Gender**								
Male	Ref.							
Female	0.63 (0.25,1.62)	0.34	3.41 (0.25,47.5)	0.36	0.71 (0.24,2.04)	0.52	0.47 (0.13,1.66)	0.47
**Years of practice**	1.00 (0.95,1.05)	0.95	1.01 (0.86,1.18)	0.92	0.93 (0.85,1.01)	0.10	0.91 (0.83,1.00)	0.91
**Duration spent with patient (min)**	0.83 (0.75,0.92)	**< 0.001**	1.15 (0.96,1.37)	0.13	1.00 (0.93,1.08)	0.94	0.93 (0.83,1.04)	0.18

*PD* Parkinson’s Disease, *GP* general practitioner, *OR* odds ratio, *aOR* adjusted odds ratio

### Departmental guidelines

Lastly, participants were asked if their department had guidelines on managing PD. Amongst GPs, 30.2% (*n* = 13) responded correctly by identifying that they do have guidelines, whereas 51.5% of geriatricians (*n* = 17) responded correctly by identifying that they do not have guidelines (*p* = 0.06).

## Discussion

This study was able to identify gaps and barriers experienced by non-neurologists in PD care in Singapore. We found that in general, geriatricians spent more time in consult with PD patients than GPs. In terms of treatment, geriatricians were also more comfortable initiating PD medications than GPs, with “unfamiliarity with the types and/or doses of the medications” being the most cited reason by both groups, though the number of years of practice of the physician did seem to increase comfort in initiating DRT. Finally, in terms of PD management practices, geriatricians were more likely to ask about falls, non-motor symptoms, and patient’s physical activity.

### Confidence and comfort in PD care

Despite both GPs and geriatricians expressing confidence in making the diagnosis of PD, GPs were less confident initiating treatment in these patients. The lower confidence levels of the GPs in the treatment of PD might be due to a confluence of factors. Some studies such as that of the Queensland paper in 2006, attribute the lower confidence levels to a lack of knowledge base [15]. After adjusting for covariates, medical specialty and duration of practice were found to have significant influence on the physician’s familiarity with types and doses of PD medication, comfort in providing PD education and whether the patients requested for neurologist confirmation of the diagnosis. Unfamiliarity with types and doses of PD medications was the most common barrier faced by both GPs and geriatricians in the care of PD patients. These results were in line with a similar study on dementia care in Singapore which suggested that education and experience is key in improving the quality of care for the patients [16]. There is a potential gap in the training of younger doctors in the area of PD management that may be overcome with years of practice and experience.

However, other factors affecting quality of care must also be considered, such as patient’s sentiments that specialists may be perceived to less likely overlook any medical-related matters [17]. In Singapore, geriatricians are seen as specialists, and hence may not encounter patients that request for a specialist (neurologist) confirmation of the PD diagnosis as frequently as GPs. This insistence on a specialist review before initiation of treatment may lead to significant delay in care and adverse outcomes for the patient [18, 19].

### Difference in management practices and styles between GPs and geriatricians

While the disparities in practices amongst physician groups might be attributed to differences in post-graduate training, geriatricians in Singapore are generally allocated more time per patient, in anticipation of more complex cases, as compared to GPs. As geriatricians spend a longer time during consults with patients, this may allow them to build stronger relationships with their patients and elicit important details during history taking [20]. A longer duration spent with patients is often recommended especially in the care of PD patients as it can improve patient satisfaction, prescribing practices, and the outcome of chronic diseases through more thorough individualized care [13, 21]. Importantly, we accounted for the potential clinical confounding effect of consult duration in our regression analysis; physician’s medical specialty remained a significant factor in management practice of PD patients.

### Gaps in practices

The questionnaire also identified areas that both GPs and geriatricians should explore more on during consultations, such as asking about taking their medication in relation to their mealtimes. Furthermore, both GPs and geriatricians can consider contacting the patient’s neurologist before adjusting patient’s PD treatment, to reduce chances of any adverse reactions [1]. For instance, abrupt withdrawal or reduction of antiparkinsonian medicines may lead to acute akinesia or neuroleptic malignant syndrome, and should be avoided [22].

### Defining and facilitating the role of non-neurologists in the care of PD patients

Plouvier et al. described GPs role in France as pertinent in the early stages of PD as knowledge of the patient’s personal context often facilitates and enhances quality of care [23]. However, that role is often limited as many GPs felt reluctant to be involved in PD care as they did not feel competent [24]. Meanwhile, geriatricians use models such as the Comprehensive Geriatric Assessment (CGA) to gain better understanding of the patient’s frailty as well as impairment of activities of daily living, to better aid their understanding in how to improve the patient’s quality of life [25–27].

Through this study, we were better able to identify certain deficits in the care of PD patients. These findings can guide the redesign of current education materials to address these deficits or the implementation of new measures to support more holistic care of PD patients.

Notably, education materials can be more focused on the types and dosage of PD medications, when to initiate treatment, and the type of history to take for PD patients (falls, non-motor symptoms, side effects of PD medications, timing in relation to meals, physical activities) [28].

One potential intervention is to provide official training and endorsement by nationally recognized bodies for neurology specialist care for community-based GPs. This can help to improve patient perceptions and provide a level of quality assurance [29, 30].

Department guidelines on best clinical practices in PD management should also be explored as an additive measure to standardize and improve quality of care. Regular audits may help to ensure that benchmarks are met. Finally, there should be seamless channels of communication between community-based care providers and neurologists to ensure the continuity of care for PD patients beyond the hospital.

A limitation of the study is that our cohort reflects only the practices of GPs and geriatricians in public institutions which have structured residency programs in place. This may underestimate the practice and knowledge gaps of PD care in the community, as private GPs and geriatricians may have even poorer access to CME and regular best clinical practice updates.

Additionally, as the questionnaire was distributed to attendees of a CME talk, the sample recruited may comprise a biased cohort who specifically sought CME in this field and therefore may not be fully representative of the GP and GRM population in Singapore. The overall survey response of 34.9% is considered above average for emailed surveys [31, 32]. The difference in response rates amongst GPs and geriatricians may introduce non-responder bias in the study. However, this is the only known study to date comparing the two populations in the management of PD.

Lastly, correction for multiple comparisons to control type I error level was not applied for the data analysis due to its small sample size and the exploratory design of the study. The overall aim of this study is to identify potential signals – where inflation in false positive rate is a lesser concern – which need to be validated in larger cohorts. Therefore, we decided on a less conservative approach to allow for a larger breadth of responses to be considered and identified. Nonetheless, even adjusting with a conservative multiple comparison approach (Bonferroni correction method), geriatricians were still more confident initiating DRT in newly diagnosed PD patients (*p* = 0.035) and were less likely to refer these patients to a neurologist (*p* = 0.01).

Despite the limitations, the study provided clear data showing certain areas of practice and knowledge gaps in the care for PD patients, which may serve to inform the design of CME for doctors in PD care. As GPs and geriatricians are more likely to be the first to encounter and diagnose a patient with PD as compared to the neurologist, it is important to better equip them with the skills and confidence to initiate treatment and monitor disease progression.

## Conclusion

This study identified key patterns between the GP and geriatrician groups in terms of their confidence levels in treating PD and the differences in their management practices. It also helped to identify gaps and differences in current practices between the two groups. Specifically, our data showed that geriatricians spend more time with patients per consult as compared to GPs, and are more comfortable with initiating DRTs as compared to GPs, with GPs citing the unfamiliarity of PD medications and doses as the most common barrier. Further studies may be conducted to identify and evaluate misconceptions that may be present in current practices to strengthen the quality of education material contents and training methodologies of medical educators. Given the chronicity of PD, clinicians should be prepared to have longer consultations with PD patients to allow for PD education, and to better facilitate discussions on how best to deliver individualized patient care. Other measures such as nationally recognized accreditation by specialist neurologist care should also be considered to increase the public’s confidence in non-neurologist to manage PD.

## Supplementary Information

**Additional file 1.** 

## Data Availability

All data generated or analyzed in this study are included in this published article.

## Abbreviations

GP: General Practitioner
PD: Parkinson’s Disease
GRM: Geriatric Medicine
CME: Continuing Medical Education
AAN: American Academy of Neurology
OR: Odds Ratio
CI: Confidence Interval
Q1 and Q3: first- and third-quartile
aOR: Adjusted Odds Ratio
DRT: Dopamine Replacement Therapy
CGA: Comprehensive Geriatric Assessment
NICE: National Institute for Health and Care Excellence
