# Supplementary material for: A cross-sectional study of knowledge and practices in the management of patients with Parkinson’s disease amongst public practice-based general practitioners and geriatricians
Source: BMC Health Serv Res. 2022 Jan 20;22:91. doi: 10.1186/s12913-022-07503-7 (PMC8780393; doi:10.1186/s12913-022-07503-7)
Supplement: Supplementary file 1 — Additional file 1. [file 12913_2022_7503_MOESM1_ESM.docx]

Appendix 1: Questionnaire

1. Gender
   1. Female
   2. Male
2. How many years have you been practicing medicine?
3. On average, how long do you spend with each patient during a follow-up consult in clinic? (round up to nearest 5 mins)
4. Are you confident of making the diagnosis of Parkinson’s disease?
   1. Yes
   2. No
5. Are you comfortable with starting dopaminergic replacement therapy in a patient whom you have diagnosed with PD?
   1. Yes
   2. No
6. If you are not comfortable, why? Choose ALL applicable options. Choose N/A if you answered ‘Yes’ above.
   1. Patients want a neurologist diagnosis of PD before starting medications
   2. Not familiar with types and/or dosages of medications
   3. Not comfortable with providing PD education
   4. N/A I am comfortable with prescribing PD medications
   5. Other: __________

1. Which PD medications are you most comfortable prescribing? Please rank 1-5, with 1 being the one you are most comfortable with. *
   1. Levodopa
   2. Dopamine agonist
   3. Amantadine
   4. Selegiline
   5. Trihexyphenidyl (Artane)

1. Do you ask about falls in patients with PD on your follow-up?
   - 1. Almost always
     2. Often
     3. Sometimes
     4. Rarely

1. Do you ask about non-motor symptoms (e.g. bowel habits, sleep, mood, cognition) in patients with PD on your follow-up?
   - - 1. Almost always
       2. Often
       3. Sometimes
       4. Rarely

1. Do you ask about side effects of PD medications (e.g. giddiness) and motor complications (fluctuations, dyskinesias) in patients with PD on your follow-up?
   - - 1. Almost always
       2. Often
       3. Sometimes
       4. Rarely

1. Do you ask your patients with PD about the timing of their medication doses in relation to meals?
   - - 1. Almost always
       2. Often
       3. Sometimes
       4. Rarely

1. Do you ask your patients with PD about their physical activities/ exercise?
   - - 1. Almost always
       2. Often
       3. Sometimes
       4. Rarely
2. Do you refer your patients with PD to a neurologist?
   - - 1. Almost always
       2. Often
       3. Sometimes
       4. Rarely

1. Do you contact your patient's neurologist (if applicable) to discuss about concerns related to his/her PD before initiating any change in management?
2. Almost always
3. Often
4. Sometimes
5. Rarely

1. Does your department have guidelines on diagnosis and management of PD?
   - - 1. Yes
       2. No
       3. I don’t know

1. What topics / questions would you like to see covered in the upcoming talk?
